# Co-existence of Functionally Different Vesicular Neurotransmitter Transporters

**DOI:** 10.3389/fnsyn.2016.00004

**Published:** 2016-02-16

**Authors:** Agnieszka Münster-Wandowski, Johannes-Friedrich Zander, Karin Richter, Gudrun Ahnert-Hilger

**Affiliations:** Institute of Integrative Neuroanatomy, Charité-Universitätsmedizin BerlinBerlin, Germany

**Keywords:** vesicular transmitter transporters, synaptic co-existence, vesicular synergy, glutamate, GABA

## Abstract

The vesicular transmitter transporters VGLUT, VGAT, VMAT2 and VAChT, define phenotype and physiological properties of neuronal subtypes. VGLUTs concentrate the excitatory amino acid glutamate, VGAT the inhibitory amino acid GABA, VMAT2 monoamines, and VAChT acetylcholine (ACh) into synaptic vesicle (SV). Following membrane depolarization SV release their content into the synaptic cleft. A strict segregation of vesicular transporters is mandatory for the precise functioning of synaptic communication and of neuronal circuits. In the last years, evidence accumulates that subsets of neurons express more than one of these transporters leading to synaptic co-release of different and functionally opposing transmitters and modulation of synaptic plasticity. Synaptic co-existence of transporters may change during pathological scenarios in order to ameliorate misbalances in neuronal activity. In addition, evidence increases that transporters also co-exist on the same vesicle providing another layer of regulation. Generally, vesicular transmitter loading relies on an electrochemical gradient ΔμH^+^ driven by the proton ATPase rendering the lumen of the vesicle with respect to the cytosol positive (Δψ) and acidic (ΔpH). While the activity of VGLUT mainly depends on the Δψ component, VMAT, VGAT and VAChT work best at a high ΔpH. Thus, a vesicular synergy of transporters depending on the combination may increase or decrease the filling of SV with the principal transmitter. We provide an overview on synaptic co-existence of vesicular transmitter transporters including changes in the excitatory/inhibitory balance under pathological conditions. Additionally, we discuss functional aspects of vesicular synergy of transmitter transporters.

## Introduction

Synaptic vesicles (SV) are key organelles for neuronal communication. They concentrate neurotransmitters produced in neural cytoplasm. Following presynaptic depolarization, cytosolic Ca^2+^ increases and SV release their content by exocytotic membrane fusion into the synaptic cleft. Fused SV are retrieved by endocytosis mainly following the clathrin-mediated pathway. In neurons, specific sets of secondary active solute transporters are responsible for filling SV with neurotransmitters. These vesicular neurotransmitter transporters (VNTs) differ from transporters in the plasma membrane with respect to energy coupling, substrate specificity and affinity. Three VNT gene families (solute carrier) comprise vesicular transporter for amines (SLC18; Schuldiner et al., 1995), for inhibitory (SLC32; McIntire et al., 1997; Sagné et al., 1997) and for excitatory amino acids (SLC17; Bellocchio et al., 2000; Takamori et al., 2000). Members account for the vesicular transport of monoamines (VMAT1 and VMAT2; SLC18A1, SLC18A2, respectively), acetylcholine (VAChT, SLC18A3), GABA/glycine (VGAT also known as VIAAT, SLC32A1), and glutamate (VGLUT1-3; SLC17A7, SLC17A6, SLC17A8, respectively). The VNTs specify the quality and quantity of SV transmitter content and thus the synaptic dynamics of neurotransmission.

With the avenue of the molecular characterization of the various VNTs, their distribution in brain areas at the cellular and subcellular level has been extensively investigated. Evidence accumulated that different types of VNTs can be expressed in the same axon terminal resulting in synaptic co-release of transmitters of opposing function like glutamate and GABA. In addition, subpopulations of SV harbor not only one type of VNT. Both, synaptic co-existence and vesicular synergy of VNTs differently affect synaptic plasticity under healthy conditions and if misbalanced may lead to various pathologies.

## Synaptic Co-existence of Neurotransmitters and VNTs

Dale’s principle that one neuron stores and releases only a single type of neurotransmitter can no longer be maintained in its strict sense. Before, neuropeptides as well as ATP have been shown to released together with acetylcholine (ACh), GABA, and monoamines (for review, see Edwards, 2007). First direct functional evidence against this principle was the demonstration of co-release of GABA and glycine from same spinal cord synapses (Jonas et al., 1998). In this system, however, both neurotransmitters share the same transporter VGAT/VIAAT.

**Figure 1:**
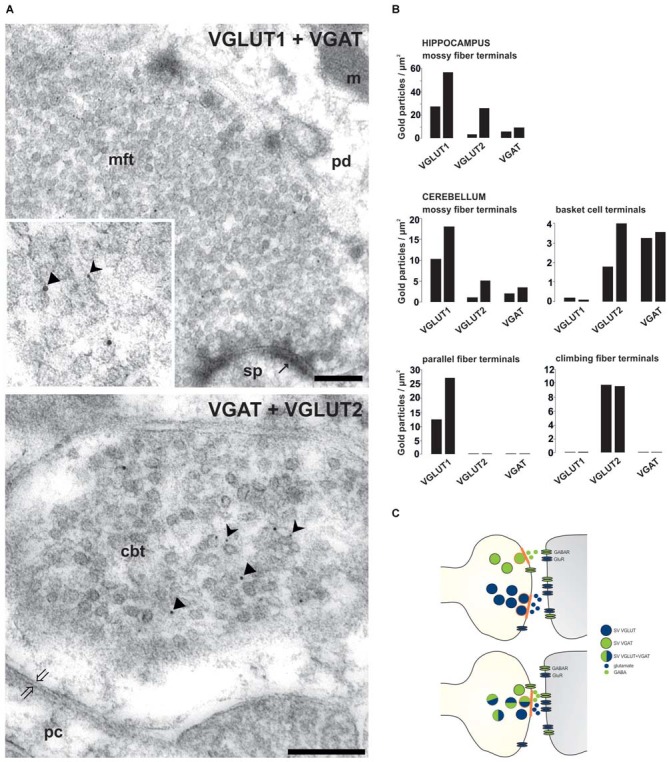
**Synapse-specific co-existence of VGLUT and VGAT. (A)** Hippocampal mossy fiber terminals (mft) of the CA3 area or cerebellar basket cell terminals (cbt) were double immunolabeled with antisera against either VGLUT1 (rabbit 5 nm gold) and VGAT (guinea pig, 10 nm gold particles) or against VGAT (guinea pig, 10 nm gold particles) and against VGLUT2 (rabbit, 5 nm gold particles), respectively (Zander et al., 2010). Distinct SV associated gold particles either indicating VGLUT are marked in insert by forked arrowheads or indicating VGAT by triangles.** (B)** Quantification of the number of gold particles staining for VGLUT1, VGLUT2 and VGAT in glutamatergic and GABAergic terminals of the hippocampal CA3 area and of the cerebellum (see Zander et al., 2010).** (C)** Scheme depicting the variations involved in synaptic co-existence of VNTs of opposing function (blue-VGLUT, green-VGAT) in the same vesicle and in different vesicle populations with distinct (above) or combined (below) release sites (orange labeled) within axon terminal. pd, pyramidal dendrite; sp, spine; pc, purkinje cell body; m, mitochondria; thin arrows indicate asymmetric contacts, thick open arrows indicate symmetric contact. Bars given in **(A)** represent 200 nm.

Studies during the last decades then demonstrated that especially glutamate could be released together with other classical neurotransmitters. Glutamate released from cholinergic spinal cord motor neurons stimulates Renshaw cells and other motor neurons (Nishimaru et al., 2005). The co-release of excitatory glutamate and inhibitory GABA neurotransmitters has been controversially discussed (Gutiérrez et al., 2003; Uchigashima et al., 2007). However, a variety of investigations including the differential synapse-specific co-existence of VGLUT1 and VGLUT2 with VGAT (Zander et al., 2010 see below) indicate that co-release of glutamate and GABA is more widespread than previously anticipated (Seal and Edwards, 2006; Chaudhry et al., 2008; El Mestikawy et al., 2011). Such co-release may not necessarily result in a direct antagonism but represents a new layer of fine-tuning synaptic networks. Following scenarios may apply to synaptic co-existence and co-release : (1) The VNTs reside on distinct populations of SVs in the axon terminal, indicating the potential for different release at distinct sites, which then activate subsets of postsynaptic receptors (Figure 1). This principle may primarily apply to giant terminals containing many transmitter release sites such as the mossy fiber terminals (MFTs; Boulland et al., 2009). (2) A spatial selective distribution of neurotransmitter receptors and their different affinities as well as variations in the type of synapses modulate the controlled activation of pre- and postsynaptic receptors (Rubio and Wenthold, 1997) depending on different transmitters released by a common site (Figure 1).

Three isoforms of VGLUTs (VGLUT1, VGLUT2 and VGLUT3) mediate the vesicular uptake of glutamate in central nervous system (CNS) neurons thereby defining glutamatergic neurotransmission. VGLUTs share salient structural and functional characteristics but differ in cellular localization and trafficking, suggesting isoform-specific physiological roles beyond their primary role of concentrating glutamate into SV (Fremeau et al., 2004; Chaudhry et al., 2008). In CNS, VGLUT1 and VGLUT2 show complementary patterns of expression mainly in subset of glutamatergic neurons (Fremeau et al., 2001; Varoqui et al., 2002). VGLUT3, by contrast, is mainly expressed in non-glutamatergic neurons characterized by other neurotransmitters (Fremeau et al., 2002; Gras et al., 2002; Takamori et al., 2002). In addition, VGLUT1 and VGLUT2 have been shown to be co-expressed in various subsets of non-glutamatergic neurons (Trudeau, 2004; Danik et al., 2005; Kawano et al., 2006), further supporting the idea that glutamate also acts as a co-transmitter.

For the inhibitory transmitters GABA and glycine only one transporter VIAAT/VGAT has been identified (McIntire et al., 1997; Sagné et al., 1997) which transports the two transmitters into SV with comparable uptake characteristics (Christensen and Fonnum, 1991). The genetic deletion of VGAT in mice finally proved, that VGAT is the only transporter for both inhibitory transmitters (Wojcik et al., 2006). VGAT is co-expressed with either the GABA-synthesizing enzymes glutamate decarboxylase (GAD65 and 67), as well as the plasma membrane GABA transporter GAT1 or the plasma membrane glycine transporter GlyT2, (Sagné et al., 1997; Chaudhry et al., 1998; Dumoulin et al., 1999; Dalby, 2003; Dufour et al., 2010), respectively. Thereby the GABAergic (Wojcik et al., 2006) or glycinergic (Gomeza et al., 2003) phenotype of neurons is defined.

Glutamate is the main excitatory neurotransmitter released from hippocampal MFTs (Crawford and Connor, 1973; Storm-Mathisen et al., 1983; Terrian et al., 1988; Bramham et al., 1990). For long, it has been known that also the inhibitory GABA transmitter is present in MFTs (Sandler and Smith, 1991; Sloviter et al., 1996). A final confirmation demands that GABA: (1) occurs in SVs of MFTs; (2) is released in response to stimulation; and (3) produces specific receptor responses. To support the idea of the dual glutamatergic-GABAergic phenotype of MFTs, we performed quantitative postembedding immunogold labeling with antibodies specific to VGLUTs and VGAT at the electron microscopic (EM) level. Postembedding immunogold EM is the method of choice to label precisely subcellular organelles, such as SV (Bergersen et al., 2008; Grønborg et al., 2010; Zander et al., 2010; Ormel et al., 2012). By this method, a synaptic co-localization of VGLUTs and VGAT in glutamatergic hippocampal and cerebellar MFTs could be demonstrated (Figure 1). The specificity of the co-existence of VGAT with VGLUT in MFTs (Figure 1) is underscored by the complete absence of VGAT immunoreactivity in other glutamatergic terminals such as cerebellar parallel (VGLUT1-positive) and climbing (VGLUT2-positive) fiber terminals identified in the same section (Figure 1). These findings provide a strong morphological evidence for synaptic co-existence of glutamate and GABA in adult glutamatergic MFTs under healthy conditions. In a similar approach, it was shown, that GABA is present together with glutamate in large MFTs and that both amino acids were associated with distinct SV (Bergersen et al., 2003, 2008). However, GABAergic synaptic transmission at MFTs requires the presence of postsynaptic GABA receptors. Indeed, GABA_A_ receptors appear to co-localizes, with glutamate alpha-amino-3-hydroxy-5-methyl-4-isoxazole propionic acid (AMPA) receptors, in postsynaptic densities in close apposition to MFTs (Bergersen et al., 2003). Their additional presynaptic contributing add to subthreshold electrical signaling in hippocampal MF (Alle and Geiger, 2007). This finding strongly suggests that MFs may convey not only a glutamatergic but also a GABAergic signal to their targets (Gutiérrez, 2005). Consistent with this hypothesis, monosynaptic GABAergic currents have been recorded in CA3 principal cells upon granule cell stimulation in the dentate gyrus in acute hippocampal slices from new-born (Safiulina et al., 2006) or juvenile animals (Walker et al., 2001; Gutiérrez et al., 2003). The evoked responses fulfil the criteria for identification of GABAergic MF inputs: strong, paired pulse facilitation, short-term frequency dependent facilitation, and sensitivity to group II and III mGluR agonists (Safiulina et al., 2006).

Several lines of evidence indicate that overshooting glutamatergic transmission play a key role in the ethology and progression of temporal lobe epilepsy (During and Spencer, 1993; Petroff et al., 2002; Cavus et al., 2005; El-Hassar et al., 2007). An increased expression of VGLUT may be important in the initiation and/or maintenance of seizures as shown in different animal models for epilepsy. Remarkably, treatment with antiepileptic drugs reduced VGLUT1 overexpression and seizure activity (Kang et al., 2005, 2006; Kim et al., 2005). In addition, a change in the balance between glutamatergic (VGLUT) and GABAergic (VGAT) expression in MFTs of the hippocampus appears to be crucial during the development of overexitation (Gomez-Lira et al., 2005).

In recent years, optogentic studies using the targeted expression of channel rhodopsin and modifications thereof yielded new insight in the mechanisms how different transmitters released from the same terminal may affect neurotransmission and behavior (Jiang et al., 2014; Trudeau et al., 2014). For example, optogenetic stimulation of arcuate hypothalamic neurons expressing diverse neuropeptides derived from pro-opiomelanocortin (POMC) leads to release of either glutamate or GABA causing fast postsynaptic actions (Dicken et al., 2012). Optogenetically labeled mesolimbic dopaminergic neurons were shown to release glutamate in the nucleus accumbens (Tecuapetla et al., 2010; Chuhma et al., 2014). In turn, habenula-derived cholinergic neurons release glutamate during brief optogenetic stimulation of their axons, and release ACh during stronger and more prolonged stimulation (Ren et al., 2011). Further examples are the regulation of spontaneous locomotor activities likely modulated by glutamate released from cholinergic interneurons in the striatum (Guzman et al., 2011). Using an optogenetic approach the relative importance of ACh released alone vs. ACh plus a co-released additional transmitter could be also distinguished. In this context, it has been shown that amacrine cells in the retina co-release ACh and GABA by distinct SV populations (Lee et al., 2010).

## Vesicular Synergy of VNTs

**Figure 2:**
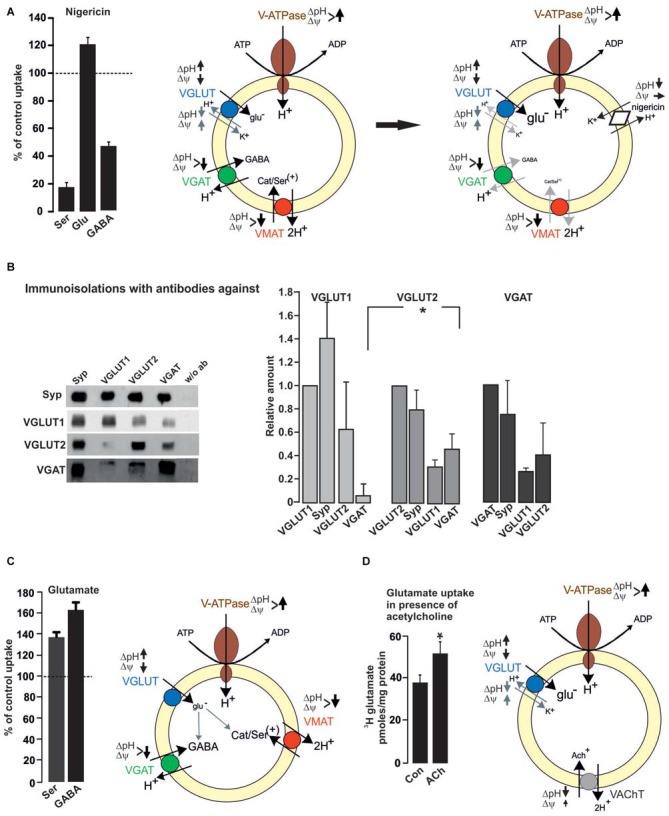
**Functional impact of vesicular co-existence. (A)** Nigericin (500 nM) increases glutamate uptake into rat brain SV, while the uptake of serotonin or GABA is inhibited. Values are presented as percent of uptake in the absence of nigericin (control) and represent mean and SD of six samples obtained in two different experiments (see also Preobraschenski et al., 2014). Nigericin promotes H^+^/K^+^ exchange thereby decreasing ΔpH with no or small effects on Δψ. This favors VGLUT activity while both VGAT and VMAT activity are reduced or dissipated, respectively. **(B)** Immunoisolation of SV populations was performed from whole rat brain with or without the antibodies indicated. A standard curve from the starting material (LS0) was run in parallel to calculate the respective protein amounts in the individual immunoisolates (Zander et al., 2010). **(C)** Glutamate increases the vesicular uptake of either serotonin or GABA into rat brain SV. Uptake in the absence of glutamate was set to 100%. Values represent the mean and SD of four samples obtained in two experiments (modified from Zander et al., 2010). Each transported glutamate molecule increases ΔpH thereby improving VGAT and VMAT activity. **(D)** Glutamate uptake into SV prepared from rat interpeduncular nucleus (IPN) is increased in the presence of acetylcholine (ACh). Values are expressed as pmoles/mg protein and were obtained from four different experiments (Frahm et al., 2015). The positively charged ACh like ${\text{NH}}_{4}^{+}$, decreases ΔpH and slightly increases Δψ, thereby improving VGLUT activity (see also Preobraschenski et al., 2014). *p < 0.05 Student’s *t*-test.

Vesicular co-existence at the synaptic level also includes vesicular synergy. Vesicular synergy involves the presence of VNTs, which differ in their dependance on the electrochemical gradient including the co-storage of transmitters with opposing function (El Mestikawy et al., 2011). Generally, all VNTs rely on the vesicular VoATPase that pumps H^+^ ions into the SV and builds up an electrochemical gradient (ΔμH^+^) rendering the vesicular lumen with respect to the cytosol positive (Δψ) and acidic (ΔpH). The various VNTs differ in their preference for either ΔpH or Δψ. VAChT and VMAT strongly depend on the ΔpH component. In contrast, VGLUT and VNUT (vesicular nucleotide transporter) appear to work best at a high Δψ. Using purified VGLUT1 reconstituted with an exogenous proton pump in liposomes, liposome-fused and native SV, the Δψ-dependance, chloride transport and H^+^/K^+^ exchange activity of VGLUT have been demonstrated (Schenck et al., 2009; Preobraschenski et al., 2014). For VGAT there is presently no agreement about the transport mechanism by which the inhibitory transmitters GABA and glycine are concentrated into SV, with a proton exchange and chloride-cotransport mechanism being discussed (Burger et al., 1991; Juge et al., 2009). The presence of different VNTs on a single SV implies that the loading of the basic transmitter is modulated by the additional transport of a second transmitter, which improves either ΔpH or Δψ depending on the combination. In this respect, the most frequent combinations involve VGLUT (one of the three isoforms) in either monoaminergic, cholinergic or GABAergic neurons. VGLUT depends on Δψ and VMAT/VAChT or VGAT exclusively or partially rely on ΔpH, respectively. The opposing effects of ΔpH for transmitter filling becomes obvious when monoamine, glutamate or GABA uptakes are performed in the presence of nigericin. Nigericin exchanges H^+^ against K^+^ thereby dissipating ΔpH with negligible effects on Δψ. Consequently, nigericin almost abolishes serotonin and partially inhibits GABA uptake but promotes vesicular filling with glutamate (Preobraschenski et al., 2014; Figure 2).

Using immunoisolation the vesicular synergy of VGLUT2 and VMAT2 has been identified in SV preparations from rat striatum (Hnasko et al., 2010). This vesicular synergy improves the monoamine filling in the presence of glutamate since each transported glutamate has to be compensated by a proton, which increases ΔpH and supports VMAT activity (Hnasko et al., 2010; Zander et al., 2010; Figure 2). Consequently, tissue levels of dopamine are decreased in the ventral striatum following deletion of VGLUT2. At the behavioral level, VGLUT2 deletion in striatal dopaminergic neurons reduces motor activity following cocaine treatment (Hnasko et al., 2010). VGLUT3 appears to occur preferentially in 5-HT neurons. These neurons also rely on VMAT2 for concentrating serotonin into SV. Comparable to the VGLUT2/VMAT2 combination in the striatum the absence of VGLUT3 causes an anxiety-related behavior in the mutants probably due to the reduced filling in subsets of serotonergic SV (Amilhon et al., 2010; see also Figure 2 and Zander et al., 2010).

All three VGLUT isoforms appear to be associated with various subpopulations of GABAergic SV. In rat cerebral cortex, immunoisolation revealed VGLUT1 but not VGLUT2 as co-existing on VGAT SV (Fattorini et al., 2009). Overall brain, vesicular synergy appears to involve VGLUT2 or VGLUT3 as dominant partners for VGAT SV (Seal et al., 2008; Grønborg et al., 2010). Immunoisolations from whole brain post nuclear fractions using either VGLUT1, VGLUT2 or VGAT antibodies support this notion with a preference of VGAT on VGLUT2 over VGLUT1 SV and* vice versa* (Zander et al., 2010; Figure 2). Irrespective of the VGLUT isoform, VGLUT/VGAT synergy may functionally improve vesicular filling with GABA in GABAergic vesicles and fine-tune the excitatory/inhibitory balance. Indeed vesicular GABA filling is enhanced in the presence of glutamate (Figure 2; Zander et al., 2010). As with VMAT, the negatively charged glutamate increases ΔpH thereby promoting VGAT activity. The functional impact for vesicular GABA loading is evident in VGLUT3 deletion mutants, which besides their deafness also suffer from rare seizures. As an additional explanation, the lack of glutamate co-release from subpopulations of GABAergic neurons may cause a hyper-synchronization of GABAergic interneurons (Ahnert-Hilger and Jahn, 2008). In the same line, the vesicular synergy of VGLUT1 on VGAT SV has been reported to fine tune an excitation/inhibition balance in cortical microcircuits (Fattorini et al., 2015). VGLUT, in particular VGLUT3 in the striatum and raphe nucleus of adult is targeted to cholinergic and serotonergic vesicles where it synergizes the action of vesicular transporters for ACh and serotonin, increasing both rate and degree of vesicle filling (Gras et al., 2008; Amilhon et al., 2010; for review El Mestikawy et al., 2011). Deletion of VGLUT3 besides the above mentioned effects in GABAergic neurons (Seal et al., 2008) causes motor hyperactivity and an increased sensitivity to cocaine explained by the reduced vesicular storage of ACh in striatal cholinergic SV (Gras et al., 2008).

A synaptic and vesicular coexistence between VGLUT1 and VAChT has been also identified in cholinergic neurons projecting from the median habenula to the interpeduncular nucleus (IPN). These neurons exhibit robust glutamatergic transmission involving AMPA and NMDA-receptor EPSCs in addition to slow nicotinic ACh-mediated currents (Ren et al., 2011). While this appears to extend the promoting effects of VGLUT on ACh filling mediated by ΔpH-dependent VAChT (see above), it was recently reported that in these neurons ACh promotes vesicular glutamate filling. Using conditionally-knocked out (cKO) mice with choline acetyltransferase (ChAT) being selectively deleted in median habenula neurons it turned out that EPSCs are reduced while the median habenula/IPN projections are perfectly equipped with VGLUT1/VGLUT2 and VAChT. The cKo–ChAT mice show reduced EPSCs and are completely insensitive to nicotine-conditioned reward and withdrawal. The findings are best explained by the lack in production and vesicular filling of ACh in the highly VGLUT expressing vesicles. ACh is a weak base, can transiently increase the luminal pH (which decreases the ΔpH) and promotes glutamate uptake (Frahm et al., 2015; Figure 2) comparable to ${\text{NH}}_{4}^{+}$ (Preobraschenski et al., 2014).

Taken together vesicular synergy represents the modulation of vesicular filling with one transmitter by changing either ΔpH or Δψ by a second transmitter (see above). As an exception, the broad co-existence between VGLUT1 and VGLUT2 (Herzog et al., 2006; Grønborg et al., 2010; Zander et al., 2010) probably does not change vesicular glutamate filling with both isoforms exhibiting similar kinetic properties. However, VGLUT1 but not VGLUT2 interacts with endophilin. This interaction changes the sorting behavior of VGLUT1 expressing SV (Voglmaier et al., 2006) and modulates their release probability (Weston et al., 2011). Thus, co-existence of VGLUT1 and VGLUT2 on the same vesicle may differentially affect endocytic retrieval modulating the persistence of VGLUTs at the plasma membrane where the transporters may be involved in Na^+^/Pi transport. Such changes in plasma membrane vs. SV associations appear to be relevant in context of a day/night cycle (Yelamanchili et al., 2006; Darna et al., 2009).

## Concluding Remarks and Future Perspectives

VNTs define the transmitter phenotype of SV and neurons and their overall expression is strictly segregated. However, there are layers of modulation at the synaptic or SV level: (1) Some neurons equip their nerve terminals with VNTs transporting transmitter of opposing or different function. This synaptic co-existence increases the functional diversity in between aminergic, GABAergic and glutamatergic neuron types. (2) Different VNTs populate the same SV in some terminals thereby modulating synaptic activity elicited by the main principal transmitter of these neurons. Examples are the vesicular synergy of VGLUT with either VGAT, VAChT, or VMAT, respectively.

Additional layers of modulation refer to the putative GABA transport by VMAT in dopaminergic neurons (Tritsch et al., 2012). Accordingly, the different endocytic retrieval of VGLUT isoforms and the transient expression of VGLUT1 at the plasma membrane also differentially shapes synaptic activity in a diurnal context. Besides glutamatergic neurons, this may also apply to neurons where VGLUT1 coexists at either the synaptic or the SV level.

## Author Contributions

AM-W, J-FZ and KR performed experiments. GA-H and AM-W wrote manuscript.

## Funding

This work was supported by the Deutsche Forschungsgemeinschaft grant AH 67/7-1*.

## Conflict of Interest Statement

The authors declare that the research was conducted in the absence of any commercial or financial relationships that could be construed as a potential conflict of interest.

## Abbreviations

ACh: acetylcholine
AMPA: alpha-amino-3-hydroxy-5-methyl-4-isoxacole propionic acid
ChAT: choline acetyltransferase
cKO: conditionally knocked out
CNS: central nervous system
EPSC: excitatory postsynaptic current
GAD65/67: glutamate decarboxylase
GAT: plasma membrane GABA transporter
GlyT: plasma membrane glycine transporter
IPN: interpeduncular nucleus
MF/MFT: mossy fiber/mossy fiber terminal
mGluR: metabolic glutamate receptor
Na^+^/Pi: transport sodium-phosphate transport
NMDA: N-methyl-D-aspartate
POMC: pro-opiomelanocortin
SLC: solute carrier
SV: synaptic vesicle
VNT: vesicular neurotransmitter transporter
VAChT: vesicular acetylcholine transporter
VGAT/VIAAT: vesicular GABA/inhibitory amino acid transporter
VGLUT: vesicular glutamate transporter
VMAT: vesicular monoamine transporter
ΔμH^+^: electrochemical gradient
Δψ: electrical gradient
ΔpH: proton gradient.
